# Towards a Sustainable European Research Infrastructures Ecosystem

**DOI:** 10.1007/978-3-030-52391-6_2

**Published:** 2020-10-30

**Authors:** Margarida Ribeiro

**Affiliations:** 1 grid.5734.5 0000 0001 0726 5157 Albert Einstein Center for Fundamental Physics, Laboratory of High Energy Physics, University of Bern, Bern, Switzerland; 2 grid.9132.9 0000 0001 2156 142X Department of Experimental Physics, CERN, Geneva, Switzerland; grid.270680.b EU, DG for Research & Innovation, Brussels, Belgium; 3 Hamburg, Hamburg Germany

## Abstract

Research Infrastructures (RIs) are of fundamental strategic importance for Europe’s global competitiveness and remain at the heart of the knowledge triangle of research, education and innovation. Of paramount importance is to coordinate the development and use of top infrastructures for data collection, management, processing and analysing while also ensuring the long-term sustainability of research infrastructures. The following essay touches on some of the key goals and available tools to develop world-class sustainable Research Infrastructures open and accessible to all researchers in Europe and beyond.

High-quality, accessible research infrastructures (RIs) are at the heart of the knowledge triangle of research, education and innovation. Whether operated and funded at regional, national or transnational level, they play a key role in advancing, exploiting and disseminating knowledge and technology while facilitating cross-sectoral international collaboration.

The European research and innovation infrastructure ecosystem is diverse in scale and scope, comprising numerous facilities and stakeholders operating at the leading edge of scientific discoveries. Moreover it provides hands-on support to local or regional stakeholder communities. Through these initiatives, the EC supports tens of thousands of researchers in academia and industry to develop innovative ideas, products and services that foster European competitiveness and help tackle some of the most pressing societal challenges that we face today.

Ensuring the availability of state-of-the-art facilities requires multi-billion Euro long-term investments across Europe. The funds come mainly from different national funding instruments. EU’s Framework Programme for R&I which amounts to a total of 4.1 billion EU (1.7 billions under FP7 and 2.4 billions under H2020) is a smaller but still useful fraction. Moreover, EU‘s Developments and Cohesion funds have also contributed to the developments of European RIs with a total amount of 18.2 billion EU in the last two programme periods (11.6 in 2007–2013 and 6.6 in 2014–2020).

Public investment in RI’s is justified given the crucial and multi-faceted role they have in advancing our knowledge around certain scientific fields, training the next generation of scientists, engineers and supporting R&D actions that enhances Europe’s innovation capacity.

Given their multiple roles, it is important to develop a modern, effective and sustainable RI ecosystem that will enable Europe to maintain its leading role in an increasingly globalized and competitive environment. For this to happen, we need to coherently integrate new infrastructures into the European landscape, plan for international governance structure, leverage the capacity to serve an international community of users and finally develop adequate long-term funding for the construction and operation, while striving to maximise their societal benefits.

This set of needs also raises the challenge to agree on the methodology for assessing RI’s impact and ensure the long-term and stable commitment of different actors involved in the different phases of a RI’s life-time.

The importance of ensuring the long-term sustainability of RIs was stressed on a number of occasions. It had already been flagged as a policy priority in the informal Competitiveness Council of July 2014. More recently, as a result of the May 2016 Competitiveness Council Conclusions [1], the Commission was invited to develop an RI long-term sustainability Action Plan, in close cooperation with ESFRI and other relevant stakeholders. The input of a stakeholder consultation process culminated in the publication of the Commission Staff Working Document “Sustainable European Research Infrastructures—A call for action” [2].

The key goals of the consultation were:

- Ensuring scientific excellence
- Attracting and training the managers, operators and users of tomorrow
- Unlocking the innovation potential of RI, Measuring socio-economic impact of RI
- Exploiting better the data generated by the RI
- Establishing adequate framework conditions for effective governance and sustainable long-term funding for RI at every stage in their life-cycle
- Structuring the international outreach of RI.

The following scheme summarizes some of the emerging themes that were described in the EC report on the Long-Term Sustainability of RIs.

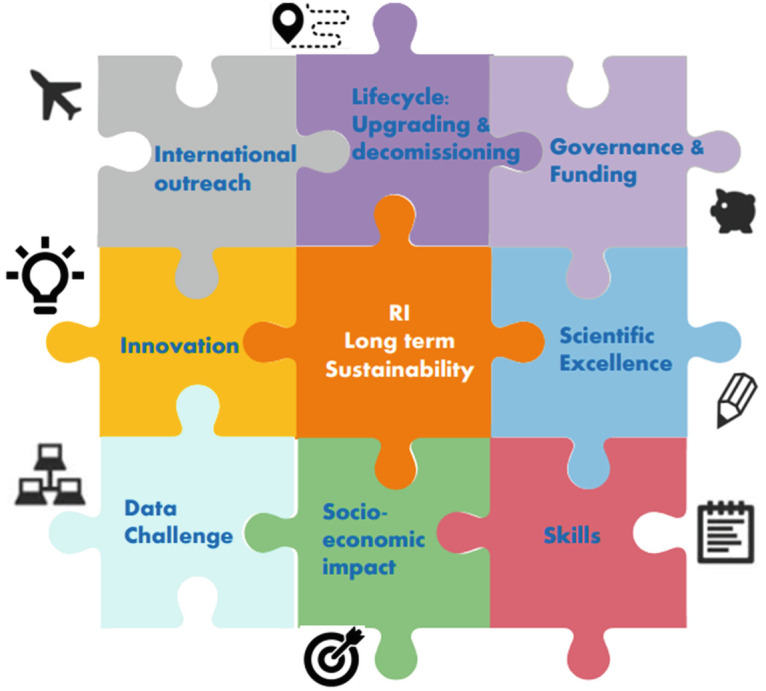



Key priority is to **ensure excellence of the services provided by the RIs.** Today it is widely accepted that excellence is the main driver for the development of RIs and this should be supported through the entire lifetime of RIs including the pursuit of research as well as the development of new technologies for and by the RI’s users. Most of the stakeholders, among which ESFRI, indicated in this respect a need to develop guidelines for standardized, effective and robust evaluation procedures of RI through independent international peer-review as an active measure to increase the widespread adoption of such instruments^1^ [3]. Knowledge, education, technology, and innovation are Europe’s main strengths and the foundation for growth and employment thus when discussing the sustainability of RIs we need to think how RIs contribute to these areas.

Another important aspect is the human capital formation that profits from RIs ecosystem. This includes the users, the managers and the operators (among other categories)^2^ [4]. When designing future RIs we need to think of the different categories who are likely to be involved in different capacities throughout their lifetime. Therefore, a challenge and a take-home message is that the right people should be in the right place and at the right time. Today about 53% of the European RI do not apply international peer review for the selection of the user projects and for attributing access; the situation needs to be rapidly addressed for present and future RIs. A way to tackle this issue would be to include a defined access policy of a RI as a requirement for funding or develop an access system based on excellency criteria. Moreover, about 21% of the RIs do not have in place an International Advisory Scientific Committee leading to the proposal that RIs should establish Technical Evaluation and Management Assessment committees.

Excellence in research requires top infrastructures for data collection, management, processing, analysing, and archiving. We need to think how we can better exploit the data from RIs and make them accessible to a wider community of users (open data, open analysis, preservation are among themes that enter this discussion). Finally, one should not underestimate the potential of RIs as hubs of innovation through the support of an ecosystem between researchers, entrepreneurs and industry around RIs.

Furthermore, one of our priorities is to encourage RIs to act as early adopters of technology and promote R&D partnerships with industry to facilitate industrial use of research infrastructures and stimulate the creation of innovation clusters. The goal is to make industry more aware of the opportunities to improve their products and co-develop technologies needed to proceed in fundamental research that could also have a market potential. Our aim is to implement policies that encourage young researchers to develop their ideas and provide the required resources that will allow them to think how these ideas can be transformed into marketable products and services. In this respect, support for RIs as hubs of interdisciplinary collaborative research in new and promising fields, can lead to greater European competitiveness, employment, and prosperity.

Two further challenges, that I would like to share with you are related to the assessment of the economic and wider societal value of RIs and the establishment of adequate framework conditions for effective governance. The first calls for the development of a standardised model, using certain data input and key indicators for identifying the socio-economic impact of RIs. A topic that also informed the organization of this workshop. As highlighted by ESFRI, national authorities and funding bodies should be explicit about the role that socio-economic impact plays in their strategy and funding decisions. Clarifying this role of RI’s will help operators and users to take appropriate action when developing future strategic plans and operating models. In that sense, periodic monitoring of societal impact should be an integral part of the regular assessment of RIs over its whole lifetime. In addition, discussions with stakeholders indicated the need to better assess the intangible investments, in quantitative terms, as today they remain rather poorly understood. This is one of the main goals of the H2020 “RI-PATHS: Research Infrastructure imPact Assessment paTHwayS” project; namely to develop a consistent, empirically implementable and holistic model at international level for analysing the socio-economic impact of research infrastructures and their related financial investments.

The second challenge is related to the need of synchronising national roadmaps with the newly established ESFRI and ERICs. Funding will be needed to support not only the construction and operation but also the transnational and virtual access of researchers and the harmonisation and improvement of the services that the infrastructures provide while an important factor that should not be neglected are the costs associated to the decommissioning of an RI at the end of its lifetime.

Finally, we need to strengthen the international dimension of pan-European RIs increasing their visibility at global scale and paving new ways for cooperation. The global dimension of societal challenges but also the complexity of new tools to push the frontiers of human understanding of the fundamental laws of nature call for better coordination and collaboration at a global level. In fundamental science, projects like the LHC that lead to the discovery of the Higgs boson or the Event Horizon Telescope that gave us the first image of a black hole clearly demonstrate this need that inform the design of future RIs.^3^ The scientific challenges addressed by European research infrastructures makes increasingly relevant their cooperation at European but also international level while exploiting synergies with research infrastructures in other world regions as well as the development of global research infrastructures.

The Research Infrastructure Programme within EU’s Horizon Europe framework for Research & Innovation aims to address these challenges by (but not only):

1. Consolidating the Landscape of European Research Infrastructures
2. Opening, Integrating and Interconnecting Research Infrastructures
3. Reinforcing European Research Infrastructure policy and International Cooperation

Each of these items breaks down into a number of specific lines of actions. I will summarize some of the key points below while for more information you can visit the EU pages for Research & Innovation [5].

Regarding the consolidation of new RIs it is important to clearly define the structure for each of the design, preparatory and implementation phases, with clear and different criteria appropriate to these levels. This will help to identify the complementarity between different funding resources while also facilitating service agreements between RIs giving the option for upgrade, merging or decommissioning of RIs.

Furthermore, it remains imperative to consider the scalability and sustainability of the European Open Science Cloud (EOSC) bringing together European, national, regional and institutional resources. EOSC’s evolution should take into account present and future needs from different research communities and the emerging technologies that could address them. This should facilitate the preservation and open access to data from RIs in compliance with the FAIR principle. Finally, in the same direction any future RI should support and be well-integrated in a pan-European research and education network.

The second pillar refers to the preparation, implementation, long-term sustainability and efficient operation of the research infrastructures identified by ESFRI and of other world-class research infrastructures, which will help Europe to respond to grand challenges in science, industry and society. The evolution of the transnational research facilities implies that RIs become elements of “supra-national innovation systems” and, in this setting, industrial players can play the role of potential supplier (of the required technologies), user and co-developer. As discussed, we need to put in place appropriate governance models and foresee a framework that will provide access of services between different RIs at regional, national and EU level. Finally, RIs managers would benefit from the development of common strategic roadmaps on the required R&D to advance certain technologies and help improve their services through partnership with industry. The innovation potential of RIs can also be expressed through the development of new services. RIs can also trigger new business models and services to policy makers.

A consultation among stakeholders has revealed the need to:

- Increase RI engagement with industry, by fostering their direct and early involvement in Advisory Boards.
- Enhance the role of intermediaries (ILO’s) and develop specific mechanisms to stimulate the commercial application of RI services and tools.
- Clarify apriori industry access rules, mainly concerning IPR regimes and procedures for accessing RI.
- Stimulate joint innovative procurement mechanisms, pre-commercial procurement and the link with Public Procurement of Innovative Solutions.
- Develop strategic roadmaps in key technologies required for the construction and upgrades of RI in close relation with EIT, KICs and KETs.

Therefore, a co-creation approach to continuously generate, scale and deploy breakthrough technologies with market and social value remains a big challenge regarding European Research & Innovation landscape.

Currently the new Research Infrastructure Programme of Horizon Europe is under development. It is among our priorities to improve synergies with other EC programmes while coordinating a Pan-European ecosystem of RIs. I believe that synergies between different funding instruments could strengthen the position of European RIs in the innovation ecosystem at EU level and to users developing new markets for key technologies.

## Footnotes

1 See ESFRI long-term sustainability WG report: https://ec.europa.eu/research/infrastructures/pdf/esfri/publications/esfri_scripta_vol2.pdf

2 See also the EC’s charter for access to research infrastructures which sets out principles and guidelines when defining access policies. https://ec.europa.eu/info/files/charter-access_en

3 Note of the Editor: this is also clearly demonstrated in the global fight against COVID-19 where numerous RIs helped to identify the structure of the new virus and in the development of new medical treatment along with the required computing resources and supercomputers for simulating the response to possible therapies as well as for tracking the evolution of the pandemic and design public health measures. A full list of EU-funded projects supporting research to tackle the COVID-19 pandemic can be found here: https://ec.europa.eu/info/research-and-innovation/research-area/health-research-and-innovation/coronavirus-research_en
